# Supplementary material for: Consumer-based actions to reduce plastic pollution in rivers: A multi-criteria decision analysis approach
Source: PLoS One. 2020 Aug 14;15(8):e0236410. doi: 10.1371/journal.pone.0236410 (PMC7428181; doi:10.1371/journal.pone.0236410)
Supplement: S1 File — (DOCX) [file pone.0236410.s001.docx]

Marazzi et al. “Consumer-Based Actions to Reduce Plastic Pollution in Rivers: a Multi-Criteria Decision Analysis Approach”

**Supporting Information**

**Table S1**. Sources included in search for consumer-led actions.

| **Source** | **Information to be reviewed** |
| --- | --- |
| Academic literature | All literature captured during the first stage of this research and flagged as being relevant to part 2 of the work during the title and abstract review stage.  N = 187 papers/grey lit/policy reports |
| NGO “Top Ten” (or similar) lists available to consumers in the UK | NGO websites/reports/toolkits scanned:   - Plastic Oceans (UK) - Ocean Conservancy (Global) - Surfers Against Sewage (UK) - Marine Conservation Society (UK) - [BBC Plastics Action](https://static-forms.tectonicinteractive.com/bbc/index-bbc-plastics.html) (call to action promoted after the BBC ‘Drowning in Plastic’ TV programme in October 2018) - UK Wildlife Trusts (UK) - City to Sea (UK) - WRAP (UK) - Friends of the Earth (UK) - Surfrider Europe [Riverine Input Project](https://www.surfrider.eu/en/story/riverine-input/conclusion/) - Earthday Network - plastics toolkit and footprint calculator - OceanChangemakers.com - Terracycle^®^ recycling initiatives (UK)   N = 14 sources |
| Books | Published books (All those related to plastic that were listed in Amazon’s top 100 environmental non-fiction list)   - [Zero Waste Home](https://www.amazon.co.uk/Zero-Waste-Home-Ultimate-Simplifying/dp/0141981768/ref=pd_sim_14_1?_encoding=UTF8&psc=1&refRID=DDVPDAF4QXE0GTQ7MRY5) (Johnson, 2013) - [How to Give Up Plastic (Greenpeace)](https://www.amazon.co.uk/How-Give-Plastic-spokesperson-anti-plastic/dp/0241363217/ref=pd_sim_14_6?_encoding=UTF8&psc=1&refRID=YQFT3DKQ1J2VVC2D26JZ) (McCallum, 2018) - [How to Live Plastic Free (MCS)](https://www.amazon.co.uk/How-Live-Plastic-Free-plastic/dp/1472259815/ref=sr_1_3?s=books&ie=UTF8&qid=1527862248&sr=1-3&keywords=plastic+free+book) (Bonaccorsi, 2018) - [Turning the Tide on Plastic](https://www.amazon.co.uk/Turning-Tide-Plastic-Humanity-Globe/dp/1409182983/ref=pd_sbs_14_3?_encoding=UTF8&psc=1&refRID=536Y6PQ3BTM59153YR09) (Siegle, 2018) - [No.More.Plastic](https://www.amazon.co.uk/No-More-Plastic-difference-2minutesolution/dp/1785039873/ref=sr_1_1?ie=UTF8&qid=1531320117&sr=8-1&keywords=no+more+plastic) (Dorey, 2018)   N = 6 books |

**Table S2**. The top ten most prevalent consumer-related plastic items found in freshwater environments in Europe (Winton et al., 2019) and the total reduction in plastic pollution to freshwater environments considering the complete adoption of the highest-ranking actions that individual consumers can take.

| **Rank** | **Plastic Item** | **Actions** | **Total Score (weighted)** | **Action rank** | **Potential tonnes saved/yr** |
| --- | --- | --- | --- | --- | --- |
| 1 | Plastic bottles  (including plastic lid or bottle top) | Use a reusable water bottle of any type | 386 | 2 | 6,741 |
| 2 | Food wrappers  (crisp packets and sweet wrappers) | Correct disposal of food wrappers | 265 | 21 | Unknown |
| 3 | Cigarette butts | Correct disposal of cigarette butts | 231 | 23 | 2,482 |
| 4 | Plastic or polystyrene takeaway containers | Use a reusable takeaway container of any type, preferably one you already own | 332 | 6 | 1,290 |
| 5 | Cotton bud sticks | Substitute plastic stick cotton buds with paper ones | 362 | 4 | 61 |
| 6 | Plastic or polystyrene cups | Use a reusable plastic cup for coffee | 331 | 7 | 1,500 |
| 7 | Sanitary items (nappies, sanitary towels, tampons and wet wipes) | Do not flush wet wipes | 306 | 10 | 3,400 |
| 8 | Smoking related packaging | Correct disposal of smoking related packaging | 203 | 27 | Unknown |
| 9 | Plastic cutlery | Use wooden cutlery when getting takeaway food | 387 | 1 | 222 |
| 10 | Plastic bags | Use a reusable cotton tote bag | 320 | 9 | 9,000 |
| **Total** |  |  |  |  | **24,696** |

**Table S3**. List of upcoming business/industry actions and innovations that were not scored within this review, but which should be reviewed and re-evaluated in the near future as they come to fruition and/or data becomes available.

| **Action** | **Item the action relates to** |
| --- | --- |
| New scheme for recycling crisp packets (Walkers^®^/Terracycle^®^) | Food wrappers |
| Substitution of non-recyclable metalized plastic film for more sustainable materials | Food wrappers |
| Existing Terracycle^®^ ‘Biscuits and Snacks Recycling Programme’ | Food wrappers |
| Plant-derived cigarette filters | Cigarette butts |
| Wood-stemmed cotton buds | Cotton bud sticks |
| Alternative sustainable materials for cutlery (other than wood) | Straws, stirrers and cutlery |

**Table S4**. Unassessed actions. List of existing or upcoming consumer actions that were not scored within this review, or received low scores, specifically due to a lack of impact data.

| **Action** | **Item the action relates to** | **Limitations** |
| --- | --- | --- |
| Use reusable sanitary towels and reusable menstrual cups | Sanitary items | No data could be sourced with which to investigate the relative carbon and water footprints of these items compared to plastic items. |
| Use organic cotton tampons with no applicator | Sanitary items |  |
| Use ‘biodegradable’ wet wipes | Sanitary items |  |
| Use ‘biodegradable’ nappies | Sanitary items |  |
| Use non-cotton reusable shopping bags e.g. recycled plastic, bamboo | Plastic bags |  |
| Use paper single-use bags | Plastic bags |  |
| Nappy collection recycling schemes such as nappicycle.co.uk in Wales | Sanitary items | No data available to score these actions as they are still niche. |
| Use toilet paper spray instead of wet wipes | Sanitary items |  |
| Reusable tampon applicator (to replace applicator tampons) | Sanitary items |  |
| **Action** | **Item the action relates to** | **Limitations** |
| Use bamboo coffee cups | Single-use cups | No data provided by bamboo cup producers to score carbon and water footprints of their cups. Keepcup LCA review includes info on bamboo cups but it is not detailed, and is not an independent study (Keepcup produce glass and plastic reusable cups, not bamboo) |
| Use non-plastic cups for all beverages | Single-use cups | Lack of data on production/use of other types of cup (i.e. non-coffee) and LCA comparisons with alternatives |
| Use non-plastic reusable bottles (aluminium, steel, glass) | Plastic bottles (including plastic lid or bottle top) | Insufficient carbon /water footprint data could be sourced, hence low scores. |
| Use non-plastic reusable food boxes (e.g. glass, metal) | Takeaway containers |  |
| Use beeswax reusable sandwich wrapping | Takeaway containers/ Plastic Bags | No data available to score this action as it is still relatively niche (despite being widely recommended by environmental NGOs). |
| Better disposal of cigarette butts, smoking-related packaging or food wrappers | Food wrappers/cigarette butts/smoking-related waste | Lack of data available on the carbon or water footprint of providing further bins, or managing the additional waste if more of these items were disposed of via landfill. |
| ‘Cigarette Waste Recycling Programme’ by Terracycle^®^ | Cigarette butts | No data available to score this action as it is still niche. |
| Use a soda stream instead of buying sparkling water or ready-made fizzy drinks | Plastic bottles | Action not considered in study |
| Buy household products in boxes rather than bottles where available e.g. laundry powder, dishwasher salt | Plastic bottles |  |

**Table S5**. Plastic reduction actions, level in the waste hierarchy and relevant UK figures on the use of plastic items (some of these figures are repeated as there are multiple actions for the same plastic item).

| **Item** | **Action** | **Waste Hierarchy** | **Volume of single-use item used in the UK / year** | **Specific reference or general source** |
| --- | --- | --- | --- | --- |
| Plastic bottles/lids | Use reusable (not single use) water bottles, supported by a refill network. This could be broken down further into plastic, glass, metal? | Reuse | 14.5 billion (all bottles) | Debbie Winton’s list |
| Cutlery | Use wooden cutlery when getting takeaway food | Rot | 16.5 billion cutlery pieces (Sherrington, 2016) | Debbie Winton’s list |
| Cutlery | Use your own reusable cutlery when getting takeaway food | Reuse | 16.5 billion cutlery pieces (Sherrington, 2016) | Wildlife Trusts |
| Cotton bud sticks | Switching to paper cotton buds | Refuse | 1.8 billion (government figure); 13.2 billion according to Sherrington (2016) | Debbie Winton’s list |
| Plastic bags | Use reusable cotton tote bags | Reuse | Large retailers in England sold 2.1 billion single-use plastic carrier bags during the year from 7 April 2016 to 6 April 2017. Note that in 2014 7.6 billion single-use plastic carrier bags in England during that calendar year, representing a 83% drop.  <https://www.gov.uk/government/publications/carrier-bag-charge-summary-of-data-in-england/single-use-plastic-carrier-bags-charge-data-in-england-for-2016-to-2017> | Debbie Winton’s list; Dorey (2018) |
| Cutlery | Use wooden stirrers | Recycle | 316 million plastic stirrers, 44 billion stirrers (Sherrington, 2016), 16.5 billion cutlery pieces (Sherrington, 2016) | Government consultation |
| **Item** | **Action** | **Waste Hierarchy** | **Volume of single-use item used in the UK / year** | **Specific reference or general source** |
| Plastic/  poly cups | Plastic reusable coffee cup (e.g. keep cup) | Reuse | 2.5 billion coffee cups thrown each year in UK | Dorey (2018) |
| Plastic bottles/lids | Get a milk delivery instead of using plastic milk bottles | Reuse | 14.5 billion (all bottles) | Marine Conservation Society (2018); Wildlife Trusts; Dorey (2018) |
| Straws/  cutlery | Use paper straws and recycle/compost | Recycle/rot | 4.7 billion plastic straws, 42 billion straws Sherrington (2016) | McCallum (2018) (p. 127) |
| Food containers | Reusable boxes (Tupperware) for lunch or takeaway items (avoid plastic/polystyrene containers) | Reuse | 5.2 billion plastic food containers | Marine Conservation Society (2018) |
| Plastic bottles/lids | Refill detergent/shampoo bottles | Reuse | 14.5 billion (all bottles) | Marine Conservation Society (2018) |
| Sanitary items | Not flushing wet wipes (behaviour change) | Reduce (those entering environment) | 3.4 billion wet wipes flushed annually UK | Sherrington (2016) (p.13) |
| Plastic bottles/lids | Choose a drink from a brand that uses recycled plastic bottles | Recycle | 14.5 billion (all bottles) | McCallum (2018) (p. 123) |
| Plastic bottles/lids | Use solid soap (instead of shower gel), shampoo and conditioner bars instead of bottles | Refuse | 14.5 billion (all bottles) | Wildlife Trusts |
| Sanitary items | Organic cotton pads | Rot | 4.3 billion | Natracare |
| **Item** | **Action** | **Waste Hierarchy** | **Volume of single-use item used in the UK / year** | **Specific reference or general source** |
| Sanitary items | Reusable nappies | Reuse | 3 billion per year in UK | McCallum (2018) (p. 133); Bonaccorsi (2018) (p. 42) |
| Plastic bottles/lids | Choose a drink from a 'more easily recyclable' container e.g. cardboard | Refuse | 14.5 billion (all bottles) | McCallum (2018) |
| Sanitary items | Menstrual cups (moon cups) | Reuse | 4.3 billion | McCallum (2018) |
| Plastic/poly cups | Glass coffee cup instead of single use (e.g. Keep cup) | Reuse | 2.5 billion coffee cups thrown each year in UK | Dorey (2018) |
| Plastic bottles/lids | Choose a drink from a 'more easily recyclable' container e.g. glass | Refuse | 14.5 billion (all bottles) | McCallum (2018) |
| Food wrappers | Improved disposal | Reduce (those entering environment) |  |  |
| Sanitary items | Reusable wet wipes | Refuse | 3.4 billion wet wipes flushed annually UK | McCallum (2018); Sherrington (2016) |
| Plastic/poly cups | Bamboo reusable coffee cup | Reuse | 2.5 billion coffee cups thrown each year in UK | Dorey (2018) |
| Sanitary items | Biodegradable wet wipes | Rot | 3.4 billion wet wipes flushed annually UK | Debbie Winton’s list |
| Cigarette butts | Improved disposal of cigarettes (behaviour change) | Responsible disposal | 44 billion cigarettes; various studies suggest that 30-92% of smokers litter | Elliott and Elliott (2018); Callaham (1995); Tidy Britain Group (1996) |
| Straws/  cutlery | Use reusable straws (bamboo/steel/glass, silicon) | Reuse | 4.7 billion plastic straws | Seas at Risk; McCallum (2018) (p. 127); BBC |
| Cigarette packaging | Improved disposal | Reduce (those entering environment) |  |  |

**Table S6**. Combined SWOT analysis on the plastic reduction actions related to the top ten plastic items (actions marked with the following letters are most related or relevant for that group of people; P: Policymakers; B: Businesses; C: Consumers; S: Scientists).

| **Action** | **Strengths** | **Weaknesses** | **Opportunities** | **Threats** |
| --- | --- | --- | --- | --- |
| **Reusable plastic bottles** | - 73% of people would like greater availability of free tap water in public spaces (P) - Refill points are becoming more publicly available through initiatives such as the Refill programme, backed by the UK water industry (P) - 65% of people would be more likely to use a reusable water bottle if tap water refills were freely available in places such as shops, airports and parks (P) - Some retailers have stopped sales of single-use plastic water bottles (B) - Reusable alternatives easy to buy (C) | - Reusable bottles are heavy and bulky. More difficult for those who do not carry a bag. Risk of spillage is a deterrent (B) - Danger people forget to carry them so they are rarely used, or people get more than they actually need, offsetting carbon/water footprint benefits (C) - Evidence that some people don’t like the taste of tap water. Varies in taste around the UK (C) - Evidence that high proportion of the British public feel uneasy or cheap asking for a tap water refill from a food service business (C) - Effectiveness rests on widespread availability of refill stations (P) | - Plastic bottles are so ubiquitous that they can be used as a starting point for discussion of wider issues related to packaging recycling and disposal (P) - Increased awareness of allowance to take empty water bottles through airport customs and provision of free tap water in departure lounges could increase uptake (P) | - Moral licensing might have unintended consequence of worsening plastic consumption elsewhere: “I’ve switched my bottle so now I don’t have to worry about recycling” (C) |
| **Improving disposal of food wrappers** | - None | - No strong incentive for consumers to comply / lack of enforcement on littering violations (P) - Will require significant behaviour change by consumers and suitable provision of bins (C) - Does not reduce the volume of food wrappers being used. They may still become litter if bins are not emptied regularly (P) | - New EU legislation will catalyse this action, as producers will be required to support the improved disposal of food wrappers (P) - New Terracycle^®^ partnerships might see more crisp and sweet wrappers recycled. Additional initiatives may be triggered (B) | - None |
| **Action** | **Strengths** | **Weaknesses** | **Opportunities** | **Threats** |
| **Improving disposal of cigarette butts** | - Pilot schemes of Hubbub “ballot bins” box demonstrated a 46% reduction in cigarette waste when trialed (B) - The same pilot showed that levels of other litter items also fell when cigarette butt litter was reduced (P) | - No strong incentive for smokers to comply (P) - High cost for local authorities to implement (additional infrastructure, bins and campaigns) (P) - Only one recent (regional) study of potential effectiveness identified (S) | - New EU legislation will require producers to reduce the plastic in cigarette filters by 80% by 2030, (which will likely prompt innovation of more sustainable filter materials) and to contribute to awareness raising and waste reduction costs (P) - Additional societal and health benefits if campaigns to reduce littering also deter smoking (P) - A Terracycle^®^ ‘Cigarette Waste Recycling Programme’ exists, but scalability and sustainability are unclear. Might see more butts recycled and additional recycling initiatives may be triggered (B) | - None |
| **Reusable takeaway containers** | - Widespread availability in shops and supermarkets (B) - Many consumers will already own one or more suitable containers (C) | - Initial higher cost to consumers if they have to purchase containers (C) - Carbon and water footprints of washing up remains unclear and will vary between households (S) - Washing facilities not always available away from home (P) - Bulky to carry around (C) - No widespread incentive schemes at present e.g. discounts (P) - Wide success would require take-back or similar schemes (P) - Difficult to implement for delivered takeaways (B) - Rarely available to buy at point of sale (B) | - EU reduction targets (25% reduction of non-recyclable materials by 2025) will likely trigger further growth in the use of reusable food containers, and the use of recyclable alternatives to single use plastic food containers (P) | - Food retailers may refuse to support reusable containers due to, for e.g. higher time/logistics involved in preparing food in different sized containers; or perceived health and safety issues (B) |
| **Action** | **Strengths** | **Weaknesses** | **Opportunities** | **Threats** |
| **Substitute plastic cotton buds with paper alternatives** | - A straight swap for consumers: functionally and economically equivalent (C) - Strong public support (C) - Near universal support among UK’s largest retailers. Many already offer them (B) - Manufacturers already produce paper-stick buds for the UK market (B) - Paper-based alternatives are cleaner to incinerate and also cleaner to produce, leading to emissions savings (P) - Potential to compost (P) | - Paper buds are heavier than plastic ones, slightly increasing fuel costs and CO_2_ emissions from transportation (B) - Slightly higher landfill disposal emission costs: relative to plastic, paper emits more CO_2e_ when placed in landfill (B) - Paper-stemmed buds are still single use and unlikely to be recycled, so may end up in landfill if not composted (P) - May still be flushed and end up as litter (P) | - Other alternatives like wood-stemmed cotton buds may lead to even greater environmental benefits over paper (wood reduces carbon emissions in production, incineration and landfill) (P) - New EU and UK legislation will catalyse this action, as plastic-stemmed buds are likely to be banned (P) | - Potential increase in littering/flushing because of perceptions that as paper/wood is eco-friendly, it doesn’t need to be put in a bin (C) - Moral licensing might have unintended consequence of worsening plastic consumption elsewhere: “I’ve switched my cotton buds so I can allow myself to buy that bottle of water” (C) - Increased pressure for raw materials to produce paper items can increase deforestation of environmentally sensitive and biodiverse areas (P) |
| **Reusable coffee cups** | - Appealing consumer product (C) - Widespread availability for consumers, sold at most major coffee outlets (B) - Incentive schemes: discounts or loyalty points for consumers who use reusable cups (B) - Some retailers now charge for a disposable cup (B) - Some coffee providers now only offer takeaway in reusable cups (B) | - Initial higher cost to consumers (but potential long-term saving) (C) - People forget them so they may be rarely used, or people get more than they need, offsetting carbon/water footprint benefits (C) - Transport costs from producer to consumer unclear and may increase carbon footprint (B) - Carbon and water footprints of washing up remains unclear and will vary between households (S) - Washing facilities not always available away from home (P) - Cups are bulky to carry, more difficult for those who do not carry a bag (C) - Risk of spillage in the bag is a deterrent (C) | - Single-use coffee cup reduction targets or a “latte levy” discussed in Parliament but initially rejected and currently only implemented voluntarily by some retailers. If legislation comes into force, will likely trigger further growth in the availability of reusable cups (P) - Opportunities for charities and other businesses to advertise or fundraise on cups (B) | - Longevity and end of life of reusable cups. Can they be easily recycled? How frequently will consumers want to ‘upgrade’ their cup? (P) - Carbon cost of some types of reusable cup, e.g. bamboo, remains unclear (S) |
| **Action** | **Strengths** | **Weaknesses** | **Opportunities** | **Threats** |
| **Not flushing wet wipes** | - The EU is planning to impose a 30% reduction target for the flushing of single use wet wipes by 2025. This would require European producers to improve labelling and consumer awareness (P) - Cost reductions for waste water management authorities (P) | - No strong incentive for consumers to change their behaviour. Widespread societal change may be challenging (P) - Additional items (e.g. nappy bags) may be required for hygienic disposal in household waste bins (P) - Additional waste diverted to landfill as wipes cannot currently be recycled, therefore increased waste management costs (P) - Labelling on packets remains poor and inconsistent (P) - Perceived inconvenience and hygiene issues by consumers (C) - No reduction in carbon or water footprint for producers or consumers (P) | - Market opportunities for product innovation into biodegradable or reusable alternatives. Defra is supporting such initiatives (P) - Bans have been announced in Washington DC (2018) and in Spain -- Balearic Islands (due in 2020) and could provide data on whether they are effective (P) | - No clear incentive in terms of price, benefit (beyond the environmental benefit), or convenience for consumers to take this action (P) |
| **Improved disposal of smoking-related packaging** | - New EU legislation means producers will be responsible for (a proportion of) the cost of awareness raising and waste disposal costs, supporting local authorities to provide improved facilities (P) | - No strong incentive for consumers to comply (P) - Will require significant behaviour change by consumers, and suitable provision of bins (C) - High cost for local authorities to implement (additional infrastructure, bins and campaigns) (P) - Does not reduce the volume of smoking-related packaging being used. May still become litter if bins are not emptied regularly (P) | - New EU legislation will catalyse this action, as producers will be required to support the improved disposal of smoking-related litter (P) - A Terracycle^®^ ‘Cigarette Waste Recycling Programme’ exists, but scalability and sustainability are unclear. Might see more smoking-related litter recycled and additional recycling initiatives may be triggered (B) | - None |
| **Action** | **Strengths** | **Weaknesses** | **Opportunities** | **Threats** |
| **Substitute plastic stirrers and cutlery with wooden alternatives** | - Wooden stirrers are comparable in price to plastic stirrers and functionally equivalent (C) - Could reduce debris on beaches in particular as wooden alternatives will decompose much quicker on their route to the beach from inland sources if they become litter (P) - Wooden cutlery and stirrers are cleaner to incinerate and cleaner to produce, leading to emissions savings (P) - Strong public support (C) - Several restaurants and fast food outlets have already switched to wooden stirrers (B) - Reduced production of plastic items is unlikely to affect UK businesses as stirrers and cutlery are predominantly imported (B) - Potential to compost (P) | - Concerns that wooden cutlery is not functionally equivalent. Some trials have been abandoned following customer complaints that spoons were not deep enough (C) - Wooden stirrers are heavier than plastic ones, slightly increasing fuel costs and CO_2_ emissions from transportation (B) - Slightly higher landfill disposal emission costs: relative to plastic, wood emits more CO_2e_ when placed in landfill (P) - Alternatives are still single use and unlikely to be recycled, so may add to landfill if not composted or incinerated (P) | - New EU and UK legislation will catalyse this action, as plastic stirrers and cutlery are likely to be banned (P) | - Small potential increase in littering because of perceptions that as paper/wood is eco-friendly, it doesn’t need to be put in a bin (C) - Moral licensing might have unintended consequence of worsening plastic consumption elsewhere: “I’ve switched my straw or stirrer so I can allow myself to buy that bottle of water” (C) - Some evidence that retailers and consumers prefer compostable plastic cutlery to wood (B) - Increased pressure to produce material for these items can increase deforestation of environmentally sensitive and biodiverse areas (P) |
| **Substitute plastic straws with paper alternatives** | - Paper-based straws are already available, and can be laminated to improve their strength (B) - Commitments have been made by major chains to switch away from plastic straws (B) - Some companies have already stopped using plastic straws (B) - Some supermarkets no longer buy plastic straws (B) - Commitments to a plastic-free alternative made by companies that use small straws with cartons (B) | - More expensive to produce than plastic straws (B) - Concerns about the quality of paper straws, with some users reporting they go ‘soggy’ and degrade while in the drink, and can affect taste (C) - Paper straws are heavier than plastic ones, slightly increasing fuel costs and CO_2_ emissions from transportation (P) - Slightly higher landfill disposal emission costs: relative to plastic, paper emits more CO_2e_ when placed in landfill (P) | - Non-plastic straws that are higher quality than paper may soon become available (B) | - Small potential increase in littering because of perceptions that as paper/wood is eco-friendly, it doesn’t need to be put in a bin (C) - Moral licensing might have unintended consequence of worsening plastic consumption elsewhere: “I’ve switched my straw so I can allow myself to buy that bottle of water” (C) |
| **Action** | **Strengths** | **Weaknesses** | **Opportunities** | **Threats** |
|  | - Paper straws are cleaner to incinerate and cleaner to produce, leading to emissions savings (P) - Strong public support (C) | - Paper straws are still single use and may end up in landfill if not recycled, composted or incinerated (P) - Not a functional equivalent for those who have medical or accessibility reasons for using a straw (C) |  |  |
| **Reusable cotton tote bags** | - Bags are light and compact and therefore convenient for consumers to carry and use (C) - Many colour/style variations available (C) - Widely available and relatively cheap to purchase in UK shops (B) - Can also be used as food produce bags (e.g. for loose fruit and vegetables) (C) | - High carbon and water footprint in production and manufacture process of cotton tote bags (P) - Need to be used hundreds of times to use the equivalent carbon and water to a disposable bag (C) - Consumers may end up with multiple bags, reducing frequency of use per bag and therefore the sustainability of the alternative (C) | - New materials could be trialed that may have lower footprints than cotton (organic cotton bags have a particularly high carbon footprint) (B) - Opportunities for charities and other businesses to advertise or fundraise on bags (B) | - Sustainable supplies of cotton or organic cotton under future climate change, given its high irrigation requirements (P) - Conversion of land to cotton farming, causing natural habitat destruction (P) |

**References (not already cited in the manuscript)**

1. Bonaccorsi L. How to Live Plastic Free: a day in the life of a plastic detox. Headline Home; 2018

Jun 7. SI

2. Callaham P. Summary of Environmental Qualitative Research: Philip Morris, 1995. Available from: <http://legacy.library.ucsf.edu/tid/aur06c00>.

1. Dorey M. (2018). No. More. Plastic.: What you can do to make a difference – the

#2minutesolution. Ebury Press.

3. Elliott, T., Elliott, L., 2018. Plastics Consumption and Waste Management 15. Available from:

<http://www.eunomia.co.uk/reports-tools/a-plastic-future-plastics-consumption-and-waste-> management-in-the-uk.

1. Johnson B. Zero waste home: The ultimate guide to simplifying your life by reducing your waste.

Simon and Schuster; 2013 Apr 9.

4. Marine Conservation Society. <https://www.mcsuk.org/media/plastic/Living_Without_Plastic.pdf>,

accessed October 10 2018).

5. Sherrington C, Darrah C, Hann S, Cole G, Corbin M. Study to support the development of measures to combat a range of marine litter sources. Report for European Commission DG Environment. 2016.

6. Siegle L. Turning the Tide on Plastic: How Humanity (and You) Can Make Our Globe Clean Again. Hachette UK; 2018 Jul 26.

7. Tidy Britain Group. [Attitudes Towards Cigarette Disposal Outdoors]. Philip Morris, 1996.

<http://legacy.library.ucsf.edu/tid/gkq47d00>.
